# Evaluating the Antioxidant Properties of the Ancient-Crop Tef (*Eragrostis tef*) Grain Extracts in THP-1 Monocytes

**DOI:** 10.3390/antiox12081561

**Published:** 2023-08-04

**Authors:** Christopher J. Cotter, Allison J. Wright, Anastasia V. Romanov, Tyler N. Graf, Eric D. Whisnant, Laura Flores-Bocanegra, Megan S. Doldron, Nicholas H. Oberlies, Zhenquan Jia, Ayalew Ligaba-Osena

**Affiliations:** 1 Laboratory of Plant Molecular Biology and Biotechnology, Department of Biology, The University of North Carolina at Greensboro, Greensboro, NC 27412, USA; 2 Department of Chemistry and Biochemistry, The University of North Carolina at Greensboro, Greensboro, NC 27412, USA; 3 Laboratory of Molecular Toxicology, Department of Biology, The University of North Carolina at Greensboro, Greensboro, NC 27412, USA

**Keywords:** *Eragrostis tef*, grain extracts, antioxidant activity, glutathione-pathway genes, phytochemicals

## Abstract

Tef (*Eragrostis tef*) is an orphan crop that is widely grown in East Africa, primarily in Ethiopia as a staple crop. It is becoming popular in the Western world owing to its nutritious and gluten-free grains and the forage quality of its biomass. Tef is also considered to have a high antioxidant capacity based on cell-free studies. However, the antioxidant activity of tef has never been validated using a physiologically relevant cell model. The purpose of this study was to investigate the antioxidant capacity of tef grain extracts using a mammalian cell model. We hypothesized that the tef grain extracts are capable of modulating the cellular antioxidant response via the modulation of glutathione (GSH) biosynthetic pathways. Therefore, we evaluated the antioxidant activity of purified tef grain extracts in the human acute monocytic leukemia (THP-1) cell line. Our findings revealed that the organic fraction of grain extracts increased the cellular GSH level, which was more evident for brown-colored tef than the ivory variety. Moreover, a brown-tef fraction increased the expressions of GSH-pathway genes, including γ-glutamate cysteine ligase catalytic (*GCLC*) and modifier (*GCLM*) subunits and glutathione reductase (*GR*), an enzyme that plays a key role in GSH biosynthesis, suggesting that tef extracts may modulate GSH metabolism. Several compounds were uniquely identified via mass spectrometry (MS) in GSH-modulating brown-tef samples, including 4-oxo-β-apo-13-carotenone, γ-linolenic acid (methyl ester), 4,4′-(2,3-dimethyl-1,4-butanediyl)bis-phenol (also referred to as 8,8′-lignan-4,4′-diol), and (3β)-3-[[2-[4-(Acetylamino)phenoxy]acetyl]oxy]olean-12-en-28-oic acid. Tef possesses antioxidant activity due to the presence of phytochemicals that can act as direct antioxidants, as well as modulators of antioxidant-response genes, indicating its potential role in alleviating diseases triggered by oxidative stresses. To the best of our knowledge, this is the first report revealing the antioxidant ability of tef extracts in a physiologically relevant human cell model.

## 1. Introduction

An imbalance between the production of reactive oxygen species (ROS) and their elimination by antioxidants results in oxidative stress [1]. Under normal physiological conditions, ROS, including superoxide anions (O_2_^•−^), hydrogen peroxide (H_2_O_2_), and hydroxyl radicals (^•^OH), act as regulators of cellular signaling pathways [2]. However, in a state of oxidative stress induced by elevated ROS concentrations or decreased antioxidant levels, ROS cause oxidative damage to nucleic acids, proteins, and lipids [3,4,5,6]. As a result, oxidative stress can accelerate the aging process and lead to a variety of pathologies, including cancer, cardiovascular diseases (atherosclerosis and hypertension), neurological diseases (Parkinson’s disease and Alzheimer’s disease), respiratory diseases (chronic obstructive pulmonary disorder), kidney disease, rheumatoid arthritis, and other chronic and acute conditions [7,8].

Organisms have evolved enzymatic and nonenzymatic antioxidant mechanisms to maintain ROS homeostasis. The enzymatic antioxidants include superoxide dismutase (SOD), catalase, peroxiredoxin, and thioredoxin reductase [1], while the nonenzymatic antioxidants include small molecules, such as glutathione (GSH), flavonoids, polyphenols, and Vitamins C, D, and E [1,7]. Of these endogenous antioxidant mechanisms, GSH is the most abundant low-molecular-weight molecule synthesized in a cell [9]. GSH is a tripeptide molecule formed from the amino acids L-glutamate, L-cysteine, and glycine that functions as an important antioxidant and cofactor for various antioxidant enzymes [10,11]. GSH primarily functions in glutathione peroxidase (GPx)-catalyzed reactions to reduce H_2_O_2_ to H_2_O [12]. Oxidized glutathione (GSSG) is then reduced back to GSH by glutathione reductase (GR), forming a redox cycle [11,12]. The biosynthesis of GSH involves two major enzymes, γ-glutamylcysteine ligase (*GCL*) and glutathione synthase, with *GCL* being the rate-limiting enzyme [12]. Decreased GSH levels have been associated with neurodegenerative disorders, pulmonary diseases, cardiovascular diseases, and other chronic conditions [11,13].

The nuclear factor erythroid 2-related factor 2–antioxidant response element (Nrf2-ARE) pathway and nuclear factor-κB (NF-κB) are the essential transcription factors (TFs) responsible for cellular responses to oxidative stress and inflammation [14]. Evidence suggests that GSH biosynthesis is modulated by the Nrf2-ARE pathway [15]. The Nrf2-ARE pathway is essential to the endogenous cellular response to ROS and electrophilic toxicants. Nrf2 regulates hundreds of genes that are involved in a multitude of cellular functions, such as cellular detoxification and the anti-inflammatory response [16]. NF-κB is involved in the pro-inflammatory-response pathway and in immune responses to infection, and it has been implicated in other basic cell functions [17]. An increasing body of literature points to the intracellular crosstalk between the Nrf2-ARE and NF-κB pathways, with new evidence that both pathways inhibit each other [14]. The interactions and mechanisms that regulate these pathways are highly complex, and many of the specifics are not well known. Aberrant regulation of both pathways has been implicated in a multitude of diseases, such as cancer pathogenesis [18]. In disease prevention strategies, the activation of the Nrf2-ARE pathway directly via its inhibitor, Kelch-like ECH-associated protein 1 (Keap1), or indirectly via NF-κB inhibition, is believed to aid in the cellular defense against disease [19]. Of particular interest in disease prevention are uses of natural-product-derived activators of Nrf2-ARE. For example, the oleanolic acid synthetic derivative 1-[2-cyano-3,12-dioxoolean-1,9(11)-dien-28-oyl]imidazolide (CDDO-Im) is a well-known Nrf2-ARE activator and has been extensively researched as a treatment strategy for inflammatory-related diseases, such as emphysema and COPD [20,21]. Krajka-Kuźniak and Baer-Dubowska (2021) summarized many natural-product-derived Nrf2-ARE- and NF-κB-pathway modulators [22].

Several dietary antioxidants, such as polyphenols and flavonoids, have been identified in various crops, including tef (*Eragrostis tef*) [23,24,25,26,27]. Tef is a cereal that is primarily grown in Ethiopia as a staple crop, and it produces the smallest grain in the world [28,29], with the grain color ranging from white to dark brown. White/ivory, red, and brown are the most popular colors. White or ivory tef has a high consumer preference and may cost 55% more than brown tef, which is more nutritious and has a stronger flavor [30,31,32]. As a staple in Ethiopian diets, tef is generally ground into flour and made into a spongy bread known as ‘injera’. Tef flour is also traditionally made into porridge or unleavened bread (‘kitta’). Due to its nutritional value and health benefits, tef is gaining popularity in the Western world as a flour alternative for use in a variety of baked goods [33,34]. Compared to other grains, both varieties of tef have high levels of essential amino acids and micronutrients, such as calcium, selenium, iron, and zinc [35,36]. Brown tef, however, contains significantly more iron, calcium, flavonoids, and phenolic acids than ivory tef [35,36,37]. Phenolic acids are well-studied antioxidants that have potential benefits for preventing cardiac disease and cancer [38,39]. Tef is also gluten-free and has a low glycemic index, making it suitable for the dietary restrictions of individuals with celiac disease (an immune reaction to gluten-containing foods) and type 2 diabetes [31,35,36,40,41,42,43].

Tef grains are also rich in polyunsaturated fatty acids (PUFAs), primarily linoleic acid (~50%) and α-linolenic acid (ALA) (~6%) [44]. Both essential fatty acids are not produced by the human body and are nutritionally very desirable because of their health benefits. PUFAs are reported to reduce the risk of diseases, such as vascular disease, arthritis, cancer, and neurological disease [45]. Linoleic acid (C18:2) is required for the normal growth of all eukaryotes [46], and it is a precursor for the synthesis of other PUFAs, such as linolenic acid and arachidonic acid. Linoleic acid has been shown to lower serum cholesterol and triglyceride levels, which helps in preventing cardiovascular diseases [47]; however, the mechanism of the therapeutic properties of tef phytochemicals, including polyphenols, flavonoids, and PUFAs, remains to be uncovered. Studies have also shown that omega-6 (ω-6) PUFAs (linoleic acid) could help in activating synergies between autophagy and the antioxidant system and greatly aid in preventing and treating multiple pathologies [48]. There are over 6000 tef germplasm collections, including over 5000 collections maintained at the Ethiopian Institute of Biodiversity [49], showing the tremendous potential to improve the content of essential phytochemicals that have various health benefits.

The antioxidant activity of tef phytochemicals has previously been reported from cell-free, small-molecule assays, such as ferric-reducing antioxidant power (FRAP), 2,2-diphenyl-1-picrylhydrazyl (DPPH), and 2,2’-azino-bis-3-ethylbenzothiazoline-6-sulfonic acid (ABTS) assays, which have indicated that tef may be a good source of antioxidants [24,25,26,43,50]; however, none of the former studies used live cell models for their antioxidant assays. While cell-free, small-molecule antioxidant assays capture information about exogenous antioxidant activity, they provide little or no information on the effect of phytochemicals on cellular endogenous antioxidants. The purpose of this study was to investigate the antioxidant capacity of tef grain extracts using a mammalian cell model. We hypothesized that the tef grain extracts are capable of modulating the cellular antioxidant response via the modulation of GSH biosynthetic pathways. Our findings revealed that active fractions of tef extracts have no cytotoxicity, but they increase the expressions of GSH-pathway genes and the intracellular GSH levels, indicating their antioxidant activity. To the best of our knowledge, this is the first report on the antioxidant ability of tef extracts in a physiologically relevant human cell model.

## 2. Materials and Methods

### 2.1. Preparation of Tef Extracts

**Figure 1:**
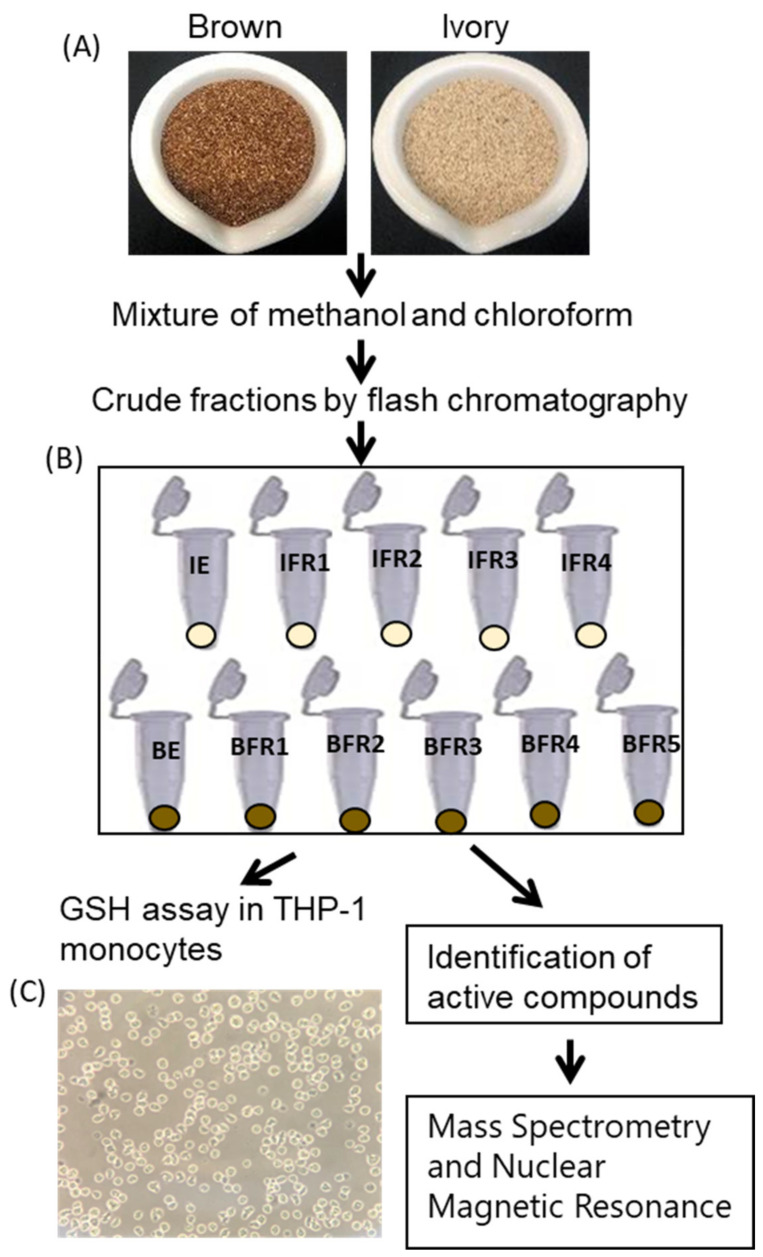
Schematic diagram showing how tef grains were extracted to prepare fractions for the antioxidant assay. (**A**) Tef seeds to be used for extraction (Note: brown and ivory are the most common tef seed colors). (**B**) Crude tef fractions obtained via flash chromatography. (**C**) Microscopic images (10×) of Human cell line (THP-1 monocytes) used for GSH assay. IE, ivory extract; IFR, ivory fraction; BE, brown extract; BFR, brown fraction.

Tef extracts were prepared as outlined (Figure 1). Brown and ivory Maskel tef (*Eragrostis tef*) varieties were purchased from the Tef Company (Boise, ID, USA). The seeds were ground using a mortar and pestle and transferred to a flask containing a 1:1 mixture of methanol:chloroform, which was soaked overnight while agitating at ~100 rpm, followed by vacuum filtration. The filtrate was transferred to a separatory funnel and a suspension was created with a 1:1 mixture of chloroform and water. The bottom layer (chloroform) was drawn off into a round-bottom flask and evaporated to dryness. The dried extract was reconstituted using a 1:1:2 methanol:acetonitrile:hexane mixture. The resulting biphasic solution was shaken and then transferred to a separatory funnel. The bottom layer (methanol:acetonitrile) was collected and evaporated to dryness under vacuum to yield a crude extract.

Crude extracts were dissolved in chloroform and adsorbed onto Celite 545 (Acros Organics) (Geel, Belgium), followed by chromatographic separation via normal-phase flash chromatography using RediSep RF Gold HP Silica columns on a Combiflash Rf 200 chromatography system (both from Teledyne ISCO) (Lincoln, NE, USA), which was monitored via an evaporative-light-scattering detector (ELSD) and photodiode-array (PDA) detector. A gradient of hexane–chloroform–methanol at a 30 mL/min flow rate and 61.0-column volumes were used to yield several fractions of the brown and ivory tef (Figure 1).

### 2.2. Mammalian Cell Culture

The antioxidant activity of the tef extracts was studied in human THP-1 monocytes purchased from the American Type Culture Collection (ATCC) (Manassas, VA, USA). The cells were cultured in RPMI 1640 media containing 10% FBS, 100 U/mL penicillin, and 100 µg/mL streptomycin at 37 °C in a 5% CO_2_/95% air environment.

### 2.3. Cell Viability Assay

To determine whether tef extracts affect cell viability, THP-1 monocytes (5 × 10^6^ cells per replicate) were incubated in RPMI 1640 media containing 5% FBS and 50 µg/mL of tef extract dissolved in DMSO (0.1% final concentration) for 24 h. Cellular GSH levels were assayed for these treated cells, as well as for cells treated with CDDO-Im (positive control, Nrf2-ARE activator) and an untreated vehicle control (V) (negative control). CDDO-Im is a triterpenoid that has been reported to increase cellular GSH levels [51]. Because the tef extracts were dissolved in DMSO, we used DMSO as another negative control. After 24 h of treatment, the cells were collected in a 15 mL Falcon tube and briefly vortexed. One milliliter of the cells was then transferred to a microcentrifuge tube and briefly vortexed. Finally, 10 µL of the cells was mixed with 10 µL of Trypan blue, 10 µL of this mixture was applied onto a hemocytometer, and the numbers of live and dead cells were counted. Cell viability was determined by dividing the number of live cells by the total number of cells counted:

(1) $$Cell\;viability\;(\%)=\frac{Number\;of\;live\;cells}{Total\;number\;of\;cells}$$

### 2.4. Glutathione (GSH) Assay

To determine whether tef phytochemicals modulate the cellular GSH level, the THP-1 monocytes (5 × 10^6^ cells per replicate) were incubated in RPMI 1640 media containing 5% fetal bovine serum (FBS) and 50 µg/mL of tef extract dissolved in DMSO for 24 h. After 24 h, the cells were pelleted via centrifugation at 1000× *g* at 4 °C for 7 min, and the resulting pellet was resuspended using 1 mL of PBS, followed by centrifugation at 5000× *g* at 4 °C for 5 min. The PBS was removed, 100 µL of 50 mM potassium phosphate buffer (pH 7.4) containing 2 mM EDTA was added to the cell pellet, and the cells were sonicated three times for 15 s. The homogenates were centrifuged at 13,000× *g* at 4 °C for 10 min. The supernatant was collected for total-protein and GSH assays. Protein concentrations were measured by adding 10 µL of the protein sample to 790 µL of Coomassie Protein Dye and measuring the absorbance at 595 nm with bovine serum albumin as the standard. The GSH concentrations were measured via the *o*-phthalaldehyde-based fluorometric method, which is specific for the determination of GSH at pH 8.0 according to Jia et al. (2008) [20]. Assays were conducted such that each biological replicate contained three technical replicates. Biological replicates were conducted in triplicate.

### 2.5. qPCR Analysis of GSH-Pathway Genes

Total cellular RNA was extracted using Trizol reagent (VWR). The RNA samples were treated with DNase I (Thermo Fisher Scientific, Waltham, MA, USA) to eliminate contaminating genomic DNA. First-strand cDNA synthesis was performed using the SuperScript III First-Strand Synthesis System (Invitrogen, Thermo Fisher Scientific, Waltham, MA, USA) according to the manufacturer’s protocol. Quantitative RT-PCR (qPCR) was performed using Applied Biosystem’s PowerUp SYBR Green Master Mix (Thermo Fisher Scientific). The relative transcript abundances of GSH biosynthetic genes (*GCLC*, *GCLM*, and *GR*) were measured in THP-1 monocytes treated with or without brown- or ivory-tef extracts. Each qPCR reaction contained 1 μL of cDNA, SYBR green Master Mix, and forward and reverse primers. Gene-specific sense and antisense primers (Table 1) were used for amplification, and the relative expressions of the genes were quantified using GAPDH and β-actin as housekeeping genes. The qPCR parameters were as follows: 95 °C for 10 min, 40 cycles of 95 °C for 15 s, and 60 °C for 1 min, followed by melting-curve analysis at 95 °C for 15 s, 60 °C for 1 min, and 95 °C for 1 s. The relative expression levels were calculated using the ΔΔCT method [52] available on QuantStudio Design & Analysis Desktop Software v1.4.3 (Appliedbiosystems, Thermo Fisher Scientific, Waltham, MA, USA). Gene expression in the untreated control cells was used as a calibrator to determine the differential gene expression in response to the tef extracts.

### 2.6. Identification of Active Compounds from Tef Extracts

Brown- and ivory-tef extracts that were identified as active and non-active based on the GSH increase in THP monocytes were subjected to mass-spectrometry analysis. High-resolution electrospray-ionization mass-spectrometry (HRESIMS) data were collected in the positive-ionization mode on a Thermo QExactive Plus mass spectrometer coupled with an electrospray-ionization source (Thermo Fisher Scientific). The HRESIMS data were individually analyzed using MZmine 2.53 to generate feature lists for each fraction. The feature lists were aligned using a custom-written Python program to align features that shared the same exact mass (within a 5 ppm variance) and an elution time within 0.1 min across all of the fractions being compared. The results of the Python program v3.10.2 were then formatted and sorted in Excel to list features that occurred primarily in the active samples but were not observed in the blanks or ivory accessions (Table S1).

Monoisotopic mass data were analyzed using ChemCalc: From Monoisotopic Mass (https://www.chemcalc.org/mf-finder, accessed 6 April 2023) to generate the molecular formulae within −5 and 5 ppm as a criterion. Prospective molecular formulae were chosen from −2 and −3 ppm and included oxygen. We input the molecular formulae into ACS SciFinder-n (https://www.scifinder-n.cas.org, accessed 6 April 2023) and the CHEMnetBASE Dictionary of Natural Products (https://dnp.chemnetbase.com/, accessed 6 April 2023) to identify candidate compounds. Known compounds identified in tef, other plants, and animal cells with antioxidant properties were used as references. Criteria such as biological significance or references from known natural products, or previously identified compounds in tef, were used to narrow down the search results.

### 2.7. Bioassay Data Analysis

Data were collected from at least three biological replicates, and the three technical replicates were analyzed via one-way ANOVA using the PROC GLM procedure. After the significant F-tests, the Tukey multiple comparison was used to separate the means (*p* < 0.01).

## 3. Results

### 3.1. Effect of Tef Extracts on Cell Viability

**Figure 2:**
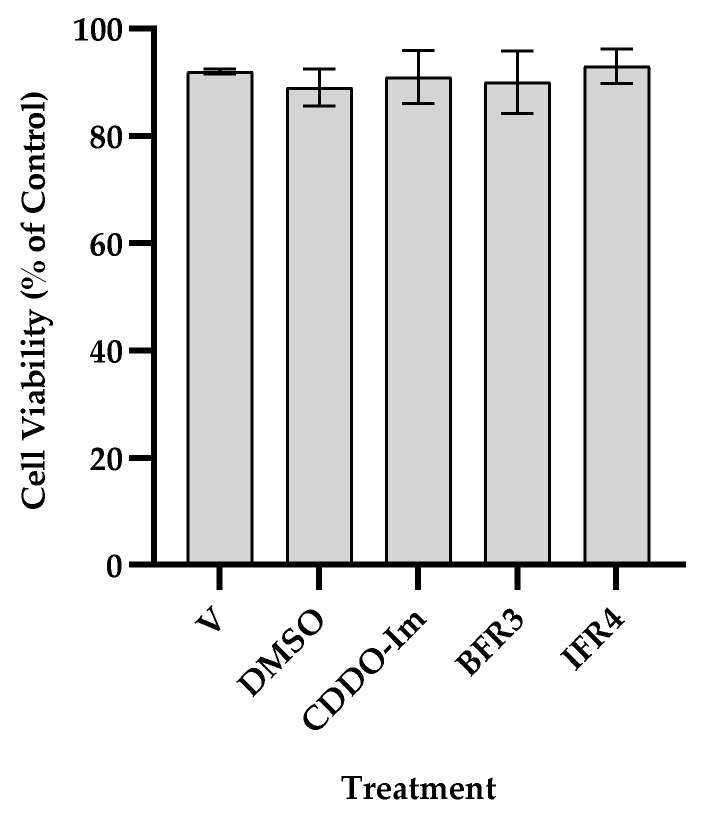
Cell viability of THP-1 monocytes in response to tef fractions. Cells were cultured without (V) or with DMSO, CDDO-Im, or 50 µg/mL of tef extracts dissolved in DMSO for 24 h, and their viability was determined by dividing the number of live cells by the total number of cells counted, as described in the Materials and Methods. Bars represent the mean and SE of three independent experiments, each replicated at least three times.

Extracts from ivory- and brown-tef grains were prepared as described in Section 2.2. Prior to testing the antioxidant activity, we tested whether the tef extracts had an effect on the cell viability. THP-1 monocytes were exposed to 50 µg/mL of extracts for 24 h, and the cell viability was determined. The cell viability, expressed as the percent of living cells per total number of treated cells, was determined via the Trypan blue staining method [20]. As shown in Figure 2, 50 µg/mL of tef extracts did not affect the cell viability. There was no difference in the cell viability between the control (untreated), DMSO, CDDO-Im, and cells treated with the tef extract. The cell viability was about 92% for the untreated control, 89% for DMSO, 91% for CDDO-Im, and 90% for brown fraction (BFR3)- and ivory fraction (IFR4)-treated cells, showing that tef extracts are not toxic to THP-1 monocytes at the concentration tested.

### 3.2. Antioxidant Activity of Tef Seed Extract

**Figure 3:**
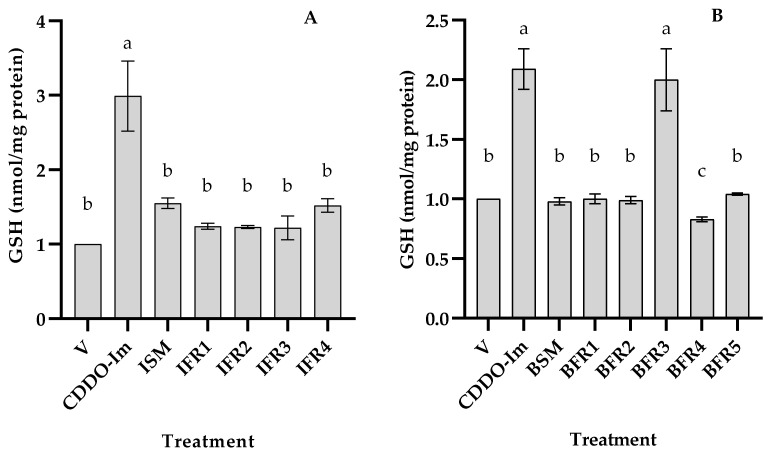
Cellular GSH in response to tef extracts and fractions. (**A**) GSH level as affected by ivory-tef grain in THP-1 monocytes. Cells were cultured without (V) or with DMSO, CDDO-Im, or 50 µg/mL of ivory-tef extract (IE) and fractions obtained via flash chromatography (IFR1, IFR2, IFR3, and IFR4). (**B**) GSH level as affected by brown-tef grain in THP-1 monocytes. Cells were cultured without (V) or with DMSO, CDDO-Im, or 50 µg/mL of brown-tef extract (BE) and fractions obtained via flash chromatography (BFR1, BFR2, BFR3, BFR4, and BFR5). Cellular GSH level was determined as described in Section 2. Bars represent the mean and SE of three independent experiments, each replicated at least three times. Bars bearing the same letter are not statistically different.

**Figure 4:**
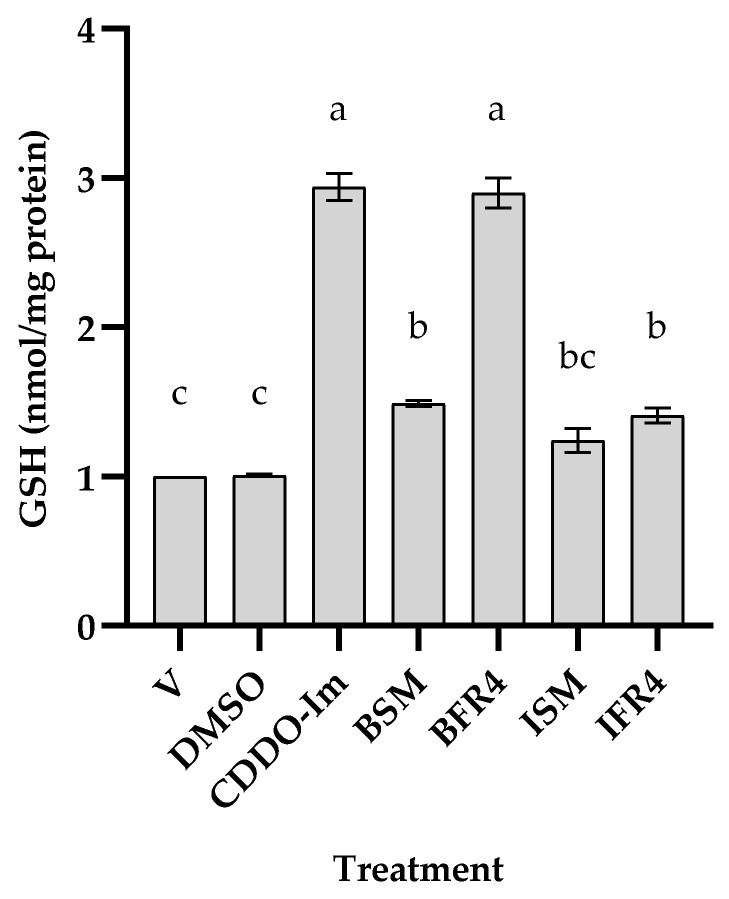
Comparison of ivory- and brown-tef extracts based on cellular GSH levels. Cells were cultured without (V) or with DMSO, CDDO-Im, and 50 µg/mL of brown-tef (BE) and ivory-tef (IE) extracts and the most active fractions of brown-tef (BFR3) and ivory-tef (IFR4) extracts obtained via flash chromatography. Cellular GSH level was determined as described in Section 2. Bars represent the mean and SE of three independent experiments, each replicated at least three times. Bars bearing the same letter are not statistically different.

To determine whether tef grains possess bioactive fractions, extracts were prepared from brown and ivory seeds and tested for antioxidant activity based on changes in the intracellular GSH content in THP-1 monocytes. The cells were incubated without or with 50 µg/mL of each tef fraction dissolved in DMSO. Five ivory-tef (ISM, IFR1, IFR2, IFR3, and IFR4) and six brown-tef fractions (BSM, BFR1, BFR2, BFR3, BFR4, and BFR5) obtained via flash chromatography were tested along with CDDO-Im (positive control, which is a known Nrf2-ARE activator) and an untreated negative control. As shown in Figure 3, the ivory-tef crude fraction (ISM-ivory starting material) and the partially purified fraction (IFR4) mildly increased the GSH content (about 1.5-fold) as compared to the negative control (V). Alternatively, the positive control, CDDO-Im, enhanced the GSH level by three-fold. Similarly, with the brown-tef fractions, a significant increase in the GSH level was observed for brown-tef fraction 3 (BFR3) as compared to the untreated control or other brown fractions (BFR1, BFR2, BFR4, and BFR5) (Figure 4). The results showed a two-fold increase in the GSH level by the brown BFR3 as compared to the control without tef extracts. The increase in the GSH level via BFR3 was comparable to that of the positive control (CDDO-Im). However, brown fraction 4 (BFR4) resulted in a small but significant decrease in the GSH level as compared to the control and other fractions (Figure 3).

**Figure 5:**
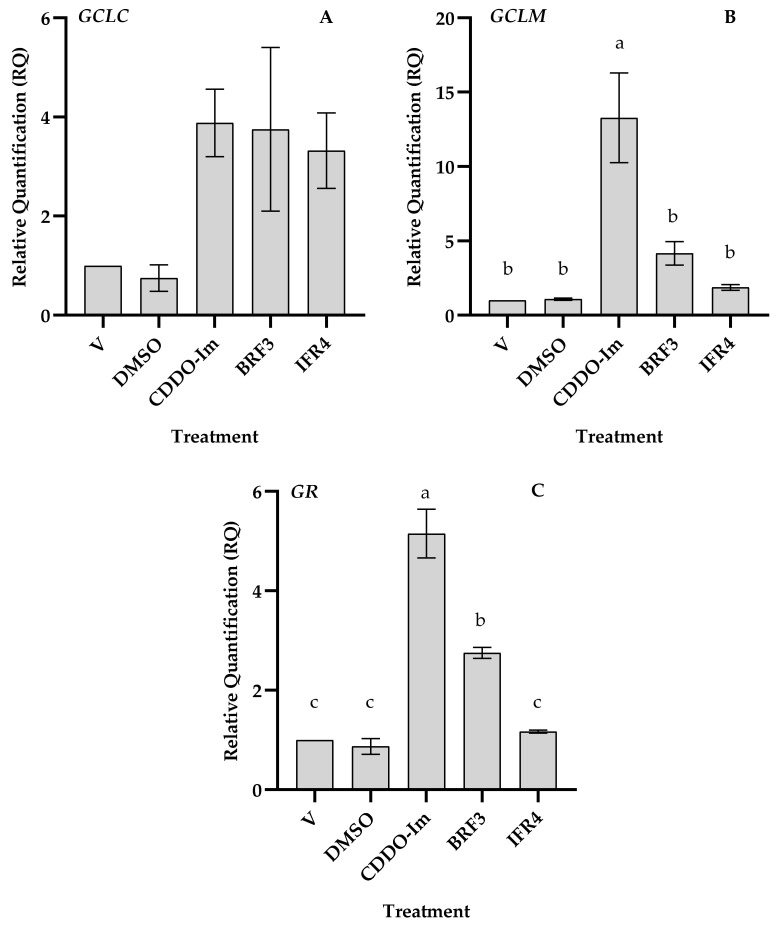
Expressions of GSH-pathway genes in THP-1 monocytes in response to tef fractions. Cells were cultured without or with DMSO, CDDO-Im, or 50 µg/mL of brown-tef (BFR3) and ivory-tef (IFR4) extracts for 24 h, and gene expression was analyzed via qPCR as described in Section 2. Bars represent the mean and SE of three independent assays, each with four replicates. Bars bearing the same letter are not statistically different. (**A**), (**B**), and (**C**) represent the relative quantification (RQ) for *GCLC*, *GCLM*, and *GR* gene expression, respectively, after treatment with V, DMSO, CDDO-Im, BRF3, and IFR4.

After identifying the active fractions of both tef varieties (IFR4 for ivory tef and BFR3 for brown tef), we treated the THP-1 monocytes with these fractions and their corresponding starting materials in the same batch of cells to determine whether there was variation in the rate of cellular GSH induction between the tef varieties. We observed that the starting material of the brown- and ivory-tef varieties increased the GSH level by 50 and 25%, respectively (Figure 5). The most active fraction of brown tef (BFR3) increased the GSH level by three-fold (i.e., 300%) as compared to the negative controls (DMSO-treated or untreated), whereas that of the ivory tef (IFR4) increased the GSH level by 45%. These findings suggest that tef seed extracts have antioxidant activity, and that there exists genetic variation among the tef accessions.

### 3.3. Effects of Tef Active Fractions on GSH-Pathway Genes

To determine whether the observed increase in the GSH level is regulated at the transcript level, we analyzed the expressions of the GSH-pathway genes γ-glutamylcysteine ligase (GCL), catalytic (GCLC), and modifier (GCLM) subunits in response to treatment with active fractions of tef extracts. Total RNA was extracted from cells treated with or without tef extracts for 24 h, and the transcript abundance was analyzed via qPCR. As shown in Figure 5, the abundance of GCLC was not affected by DMSO, whereas treatment of the THP-1 with the active fractions of brown-tef (BFR3) and ivory-tef (IFR4) extracts increased the GCLC transcript abundance by about four-fold. There was no marked difference between the increases in the GCLC gene expression with the CDDO-Im, brown-, and white-tef fractions. Treatment of the THP-1 monocytes with DMSO had no marked effect on the expression of GCLM, while the BFR3 and IFR4 extracts enhanced the GCLM gene expression by about four- and two-fold, respectively (Figure 5). The expression of GCLM via CDDO-Im was significantly higher than that of both tef fractions. We also analyzed the expression of glutathione reductase (GR), an enzyme that uses NADPH to reduce glutathione disulfide (GSSG) back to GSH [11,12,53]. The results showed that the active fraction of the brown-tef extract increased the expression of GR by about three-fold, while the GR gene expression was not affected by the ivory-tef fraction or the negative control, DMSO (Figure 5). As observed for GCLM, the expression of GR was significantly higher for cells treated with CDDO-Im as compared to the two tef fractions.

### 3.4. Identification of Active Compounds in Tef Extracts

Because the active fractions, particularly those of the brown-tef extracts, increased the cellular GSH levels and expressions of GSH-biosynthesis genes in THP-1 monocytes, we subjected the samples (Figure 1) to mass spectrometry (MS) to identify the compounds that are likely responsible for the increased antioxidant activity. We identified thirty-eight unique compounds that were detected only in the active samples and not in the fractions that showed no antioxidant activity or the blank sample.

**Figure 6:**
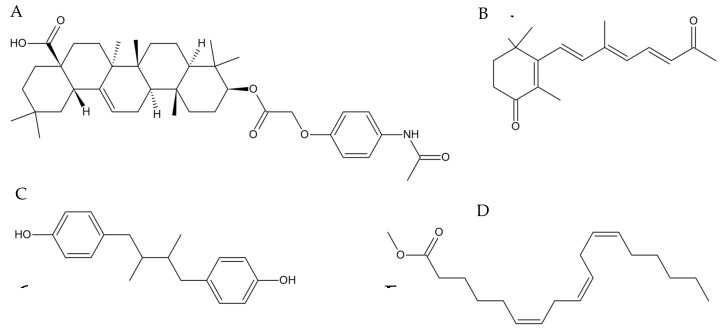
Tef phytochemicals uniquely identified in active tef fractions: (**A**) (3β)-3-[[2-[4-(acetylamino)phenoxy]acetyl]oxy]olean-12-en-28-oic acid, (3β)); (**B**) 4,4′-(2,3-dimethyl-1,4-butanediyl)bis-phenol or 8,8′-lignan-4,4′-diol; (**C**) 4-oxo-β-apo-13-carotenone; (**D**) γ-linolenic acid (methyl ester). The THP-1 monocytes were treated using chromatographically purified tef grain extracts, and changes to intracellular GSH levels were observed. Tef grain fractions that increased GSH levels were deemed active and later analyzed via mass spectrometry. Thirty-eight unique features, not observed in the inactive fraction, were identified using an online database, and these were narrowed down to four candidate compounds.

Using the chemical databases SciFinder-n (https://www.scifinder-n.cas.org, accessed 6 April 2023) and the CHEMnetBASE Dictionary of Natural Products (https://dnp.chemnetbase.com/, accessed 6 April 2023), we identified several compounds in the active tef fractions, including 4-oxo-β-apo-13-carotenone (C_18_H_24_O_2_, CAS registry number 146583-04-2), γ-linolenic acid (methyl ester) (C_19_H_32_O_2_, CAS registry number 16326-32-2), 4,4′-(2,3-dimethyl-1,4-butanediyl)bis-phenol (also referred to as 8,8′-lignan-4,4′-diol) (C_18_H_22_O_2_, CAS registry number 56319-00-7), and (3β)-3-[[2-[4-(Acetylamino)phenoxy]acetyl]oxy]olean-12-en-28-oic acid (C_40_H_57_NO_6_, CAS registry number 2649473-28-7) (Figure 6, Table 2). Our search identified a number of compounds with the same chemical formulae as those listed here (Table S2). However, we selected those chemicals that have been implicated in the oxidative-stress pathway directly or indirectly and discuss them below.

## 4. Discussion

### 4.1. Observed Cell Activity with Respect to the Mass-Spectrometry Analysis

The antioxidant activity of tef phytochemicals has been reported by some researchers from studies based on non-cell-based assays, including ABTS, DPPH, ferric-reducing antioxidant power (FRAP), and oxygen radical absorbance capacity (ORAC) assays [25,43,49]. Antioxidant activity may be influenced by several factors, including the test system, type of phytochemical, method of preparation, etc. Non-cell-based assays primarily evaluate the antioxidant activity of bound and free flavonoids and polyphenols in tef grains. For example, Kotásková et al. (2015) reported that free phenolic fractions showed higher antioxidant activity using both the ABTS and DPPH methods [25]. Similarly, Rocchetti et al. (2019) reported that phenolic extracts from tef possess higher antioxidant activity than other cereal and pseudocereal flours, including black and red quinoa and red and white sorghum, based on FRAP and ORAC values [50]. These prior studies have also shown higher antioxidant activity for brown-tef varieties as compared to white/ivory varieties [25,43], suggesting the presence of genetic variation in the antioxidant activity. Our findings are consistent with previous studies. Some tef fractions obtained via flash chromatography showed antioxidant activity based on an increase in the cellular GSH levels in THP-1 monocytes (Figure 3, Figure 4 and Figure 5). This increase in the GSH levels was significantly higher for brown-tef extracts as compared to extracts from white/ivory tef (Figure 5).

### 4.2. Features Identified from the Mass-Spectrometry Analysis

As described in the ‘Materials and Methods’, we identified 38 unique features in the bioactive tef fractions that were not observed in the methanol blanks or extracts from the ivory-tef accession (Table S1). From these, the compounds were narrowed down to four candidates, including 4-oxo-β-apo-13-carotenone, γ-linolenic acid (methyl ester), 4,4′-(2,3-dimethyl-1,4-butanediyl)bis-phenol (also referred to as 8,8′-lignan-4,4′-diol), and (3β)-3-[[2-[4-(acetylamino)phenoxy]acetyl]oxy]olean-12-en-28-oic acid.

It is known that brown-tef seeds contain higher levels of C18:3 and polyunsaturated fatty acids (PUFAs) [44,54], which are known to be essential phytochemicals. Tef grains contain primarily linoleic acid (~50%) and α-linolenic acid (ALA) (~6%) [44]; both essential fatty acids are not produced by the human body and are nutritionally very desirable. The γ-Linolenic acid (GLA) detected in the active fraction of brown tef in this study is very similar to ALA. The involvement of GLA in oxidative stress has not been studied. However, it has been reported that GLA inhibits intracellular inflammatory responses by regulating NF-κB activation [54]. It has also been shown to restore cellular GSH in RAW 264.7 macrophages that were suppressed by lipopolysaccharide (LPS), an activator of the pro-inflammatory TF NF-κB [55]. This suggests that GLA may inhibit the activation of the pro-inflammatory pathway by inactivating NF-κB through the suppression of the signal transduction pathway ERK and the suppression of oxidative stress [54,56]. Previously, it was established that Nrf2 activation regulates the expressions of a wide variety of anti-inflammatory products. Many plant-derived anti-inflammatory compounds activate Nrf2 via NF-κB inhibition [22]. It is also possible that GLA may play a role in the observed increase in GSH through indirect Nrf2 activation and the increased expressions of GSH biosynthetic genes.

Our analysis also identified an oleanolic acid (OA) analogue, (3β)-3-[[2-[4-(acetylamino)phenoxy]acetyl]oxy]olean-12-en-28-oic acid, in the active brown-tef fractions. OA has traditionally been used in Chinese medicine to treat liver disease and has received increased interest for its potential antioxidant effects. Oleanolic acid is a naturally derived triterpenoid compound reported in over 120 plant species [19,57]. Natural and synthetic OA derivatives have been found to modulate multiple pathways, including NF-κB and Nrf2 [19]. Its synthetic derivative, CDDO-Im, is a known Nrf2 activator and generator of potent anti-inflammatory responses [19]. CDDO-Im was used as a positive control for the GSH assay discussed above. Wang et al. (2010) reported that OA acts primarily as a modulator of the anti-inflammatory response and, to a lesser extent, as a direct antioxidant [57]. In ROS-induced QZG cells, OA was found to ameliorate the intracellular GSH concentrations compared to non-OA-treated cells [57]. Moreover, the expressions of key antioxidant enzymes mediated by Nrf2 have been shown to increase in cells treated with OA compared to controls, indicating that the OA analogue detected in the active fraction might have contributed to the observed increase in the GSH levels and expressions of GSH biosynthetic genes in the THP-1 monocytes studied here.

In the active tef fraction, we also identified 4-Oxo-β-apo-13-carotenone. Carotenoids can be transformed into smaller apocarotenoids via carotenoid cleavage dioxygenases (CCDs) [58]. Various cleaving reactions of β-carotene lead to 4-Oxo-β-apo-13-carotenone, although the exact mechanism remains unknown [59]. Lycopene, a carotenoid, was found to transactivate the expressions of reporter genes fused with the ARE sequence in transfected cancer cells. Other carotenoids, such as β-carotene, had smaller effects on ARE activation. Lycopene has been shown to increase the expressions of two phase II enzymes, NQO1 and GCS, and to initiate the nuclear translocation of the Nrf2 TF, confirmed via luciferase assay [60]. Other apocarotenoids, such as bixin, have been found to activate Nrf2 via interactions with key cysteine sensor residues of Keap1, generating a strong antioxidant-expression response in keratinocyte cells [61]. Although 4-Oxo-β-apo-13-carotenone, lycopene, and bixin are produced via similar cleavage reactions and share structural commonalities, we can only speculate about the potential role of 4-Oxo-β-apo-13-carotenone in Nrf2 activation and the antioxidant system.

The 4,4′-(2,3-dimethyl-1,4-butanediyl)bis-phenol (herein referred to as 8,8′-Lignan-4,4′-diol), which was also detected in the active fraction, is a lignan structurally comparable to 20-hydroxy dihydroguaiaretic acid, extracted from underground parts of *Saururus chinensis* [62]. It has been reported that 20-Hydroxy dihydroguaiaretic acid exhibits low-density-lipoprotein (LDL) antioxidant activity and free-radical DPPH scavenging activity [62]. Lignans have been known to exhibit antioxidant activity due to their phenolic structure. Lignans can act as direct antioxidants and aid in mitigating free-radical damages [63]. Sauchinone, a bioactive lignan, was reported to protect hepatocytes against acetaminophen-induced toxicity by activating Nrf2 [64]. An analogue of 8,8′-Lignan-4,4′-diol, nordihydroguaiaretic acid (NDGA), was found to be a potent antioxidant and activator of Nrf2 in cerebellar granule neurons [65]. They both are a class of lignans called dibenzyl butane lignans, differing in two additional hydroxy groups on NDGA that are not present in 8,8′-Lignan-4,4′-diol. The same study found that NDGA increased the expressions of Nrf2-mediated genes and identified increased Nrf2 nuclear translocation via a luciferase assay [64]. We did not study whether 8,8′-Lignan-4,4′-diol directly activates Nrf2, and we did not find any report on this topic; however, given its structural relationship to NDGA, 8,8′-Lignan-4,4′-diol may also activate the Nrf2-mediated antioxidant response. Further study is needed to determine whether 8,8′-Lignan-4,4′-diol is directly involved in the antioxidant pathway.

### 4.3. Expressions of GSH Biosynthetic Genes and Cellular GSH

To determine whether increased cellular GSH is due to its biosynthesis, we analyzed the expressions of key GSH-pathway genes, including GCLC, GCLM, and GR. As shown in Figure 5, treatment of the THP-1 monocytes with tef extracts increased the gene expression of the rate-limiting enzyme of glutathione biosynthesis (GCLC) by about three-fold, as observed for the positive control, CDDO-Im. The active fractions of both the brown and ivory tef enhanced the expression of GCLC, but to a lesser extent compared to CDDO-Im, which increased the transcript level by at least 10-fold. The most active brown fraction (BFR3) enhanced the GCLM expression by more than four-fold. GCLC plays a critical role in maintaining GSH homeostasis, and its expression level is usually consistent with the cellular GSH concentration [66]. The expression of the GCL modifier (GCLM) subunit was enhanced by the active fraction of brown tef by about five-fold, while its expression was only induced by two-fold in cells treated with the ivory fraction. These findings are consistent with the GSH level, which was higher in cells treated with the brown fraction than the ivory fraction, showing that lower GSH levels in cells treated with the ivory-tef fraction could be due to lower GCLM activity, leading to a lower rate of GSH biosynthesis. Glutathione is a vital antioxidant, the cellular level of which depends on the rate of biosynthesis and changes in the ratio of intracellular reduced and oxidized forms of GSH [67]. Therefore, we also assayed the expression of GR, which regulates GSH homeostasis by catalyzing the reduction of GSSG to GSH. As shown in Figure 5, the expression of GR was increased five-fold in cells treated with the brown-tef fraction, whereas the extract from ivory tef had no effect on the GR expressions. Given that the brown-tef extracts enhanced the expressions of the GSH biosynthetic (GCLC and GCLM) and GSH homeostasis (GR) genes, increased cellular GSH via tef extract is likely due to increased biosynthesis and the intracellular conversion of GSSG to GSH.

It has been reported that the expressions of GCLC and GCLM are regulated by the Nrf2–antioxidant response element (ARE) pathway [15]. The Nrf2 pathway is the main pathway that is activated via ROS production, and it is reported to induce about 250 target genes, including GCLC, GCLM, GSH synthase, NQO1 (NAD(P)H Quinone Dehydrogenase 1), HO-1 (Heme oxygenase 1), superoxide dismutase (SOD) and catalase (CAT), and enzymes of the glutathione–thioredoxin system (GR, GPx, and GST) [68]. Under normal conditions, Nrf2 is bound to dimeric Kelch-like ECH-associated protein 1 (Keap1), is ubiquitinated by Cul3/Rbx, and is targeted for degradation [69]. However, in response to reactive oxygen species (ROS), electrophiles, and many phytochemicals [22,70,71,72], Nrf2 migrates to the nucleus and binds to a *cis*-acting DNA promoter sequence, termed the antioxidant response element (ARE), which allows the transactivation of a group of cytoprotective genes [73,74]. Tef phytochemicals may induce the activation of Nrf2, which triggers its migration to the nucleus. Nrf2 may then bind to the ARE and regulate the expressions of various target genes, including genes of the antioxidant pathway. This may lead to the biosynthesis of antioxidant molecules, such as GSH. However, we cannot rule out that enhanced cellular GSH by tef extract may be regulated via a Nrf2-independent-pathway mechanism.

### 4.4. Observed Limitations and Future Directions

We are the first to report on the antioxidant effects of tef extracts using a physiologically relevant cell-based model, and we are in the process of elucidating the underlying physiological and molecular mechanisms in which tef may act as a powerful dietary source of antioxidants. As a result, the discussions and conclusions of this study may seem speculative to some extent. One challenge is that the identification of potential compounds that contribute to these antioxidant activities is only tentative at this time. We have not isolated these compounds yet or verified their activities individually; such studies are planned for the future. The feature-based analysis is powerful in that it suggests the compounds to target. However, the identifications are tentative and based on matches to literature values. It is possible that other compounds that have not been published in the literature yet could also contribute to the observed biological activity. For example, some features that returned molecular formulae did not yield solid hits when searched against two separate chemical databases used in the analysis. Alternatively, the molecular formulae returned many potential compounds that were screened extensively, and only those that were structurally relevant and validated in the literature for bioactivity, such as antioxidants, were selected. Therefore, there is a need to purify the bioactive compounds, quantify these compounds, and test their potential toxicity and antioxidant activity against pure or synthetic products.

Furthermore, our study revealed that tef extracts increase the expressions of GSH-biosynthetic-pathway genes, which are regulated by the TF Nrf2, which is the master regulator of the antioxidant pathway. However, it remains to be elucidated whether the observed increases in cellular GSH are via the activation of Nrf2 or a mechanism that is independent of the Nrf2 antioxidant pathway. Regardless, we believe that the observed modulation in GSH biosynthesis can be attributed, at least in part, to the bioactive phytochemicals identified in the tef extracts via the mass-spectrometric analysis.

## 5. Conclusions

This study revealed that tef grain extracts increased cellular GSH levels. This response was more pronounced in the brown-tef extracts compared to the ivory-tef accession analyzed in this study. This appears to be due to the higher phytochemical content in brown tef compared to ivory tef. The most active fraction of brown tef also increased the expressions of some GSH-pathway genes, which are known to be directly regulated by Nrf2 and indirectly by NF-κB. Further study is needed to identify the molecular mechanism underlying enhanced GSH accumulation in vitro using physiologically relevant model cells, as well as in vivo using model animals. Underutilized or orphan crops such as tef are untapped sources of essential food constituents, such as bioactive polyphenols, flavonoids, PUFAs, etc., which could alleviate oxidative stress and associated diseases. We are conducting an ongoing study to elucidate the physiological and molecular mechanisms underlying the increased cellular GSH in response to treatment with tef extracts. Studies are also ongoing to determine the range of genetic variability among tef germplasm in antioxidant activity.

## Figures and Tables

**Table 1:** Gene-specific sense and antisense primers used for qPCR.

Gene Target	Gene ID	Amplicon Size	Forward Primer	Reverse Primer
*GCLM*	NM_002061 V4	246 bp	5’-CTCCCTCTCGGGTCTCTCTC-3’	5’-ATCATGAAGCTCCTCGCTGT-3’
*GCLC*	NM_001197115	182 bp	5’-ACCATCATCAATGGGAAGGA-3’	5’-GCGATAAACTCCCTCATCCA-3’
*GR*	NM_000637 V5	205 bp	5’-CAGTGGGACTCACGGAAGAT-3’	5’-AAACCCTGCAGCATTTCATC-3’

**Table 2:** List of features identified and characterized from the active fraction of brown-tef extract.

Feature *	[M+H]^+^ *m/z*	Calculated *m/z*	Molecular Formula	Potential Compounds
2	273.1848	272.1769	C_18_H_24_O_2_	4-Oxo-β-apo-13-carotenone
18	271.1692	270.1612	C_18_H_22_O_2_	4,4′-(2,3-dimethyl-1,4-butanediyl)bis-phenol
31	648.4251	647.4172	C_40_H_57_NO_6_	(3β)-3-[[2-[4-(Acetylamino)phenoxy]acetyl]oxy]olean-12-en-28-oic acid
36	293.2478	292.2398	C_19_H_32_O_2_	γ-Linolenic acid, methyl ester

* Numbering of features refers to the full supplemental table (Table S2).

## Data Availability

The data presented in this manuscript are included in the article and two supplementary tables.

## Supplementary Materials

The following supporting information can be downloaded at https://www.mdpi.com/article/10.3390/antiox12081561/s1, Table S1. Feature lists of HRESIMS data of tef fractions individually analyzed using MZmine 2.53. The feature lists were aligned using a custom-written Python program. The results of the Python program were then formatted and sorted to list features that occurred primarily in the active samples. Table S2. List of compounds that were uniquely detected in brown-tef fraction compared to ivory fraction with their chemical formulae and predicted feature names.
